# Parkinson’s disease with a typical clinical course of 17 years overlapped by Creutzfeldt–Jakob disease: an autopsy case report

**DOI:** 10.1186/s12883-021-02504-1

**Published:** 2021-12-10

**Authors:** Shin-ichiro Kubo, Tomoyasu Matsubara, Takeshi Taguchi, Renpei Sengoku, Atsuko Takeuchi, Yuko Saito

**Affiliations:** 1 Department of Neurology, Eisei Hospital, 583-15 Kunugidamachi, Hachioji, Tokyo, 193-0942 Japan; 2 Department of Neuropathology (the Brain Bank for Aging Research), Tokyo Metropolitan Geriatric Hospital and Institute of Gerontology, 35-2 Sakae-cho, Itabashi-ku, Tokyo, 173-0015 Japan; 3 Department of Neurology, Tokyo Medical University Hachioji Medical Center, 1163 Tatemachi, Hachioji, Tokyo, 193-0998 Japan; 4 Department of Neurology, Daisan Hospital, The Jikei University School of Medicine, 4-11-1 Izumihoncho, Komae, Tokyo, 201-8601 Japan; 5 Department of Neurological Science, Tohoku University Graduate School of Medicine, 2-1 Seiryo-machi, Aoba-ku, Sendai, 980-8575 Japan

**Keywords:** alpha-synuclein, Creutzfeldt–Jakob disease, Lewy bodies, Parkinson’s disease, prion

## Abstract

**Background:**

Late-stage Parkinson’s disease (PD) often presents with neuropsychiatric symptoms such as dementia, psychosis, excessive daytime sleepiness, apathy, depression, and anxiety. However, neuropsychiatric symptoms are the cardinal features of Creutzfeldt–Jakob disease (CJD), raising the possibility that CJD may be an overlooked condition when it accompanies late-stage PD.

**Case presentation:**

We describe a female autopsy case of PD with a typical clinical course of 17 years, in which CJD overlapped with PD during the final year of the patient’s life. The patient died aged 85 years. Neuropathological features included widespread Lewy body-related α-synucleinopathy predominantly in the brainstem and limbic system, as well as the typical pathology of methionine/methionine type 1 CJD in the brain.

**Conclusions:**

Our case demonstrates the clinicopathological co-occurrence of PD and CJD in a sporadic patient. The possibility of mixed pathology, including prion pathology, should be taken into account when neuropsychiatric symptoms are noted during the disease course of PD.

**Supplementary Information:**

The online version contains supplementary material available at 10.1186/s12883-021-02504-1.

## Background

Parkinson’s disease (PD) is the second most common neurodegenerative disease after Alzheimer’s disease, affecting more than 2% of people aged 65 and over [1]. The clinical features of PD consist of motor dysfunction, including bradykinesia, resting tremor, and rigidity, as well as non-motor symptoms, such as sleep disorders, mood disorders, sensory symptoms, olfactory dysfunction, dysautonomia, cognitive impairment, and dementia. The pathological hallmarks of PD are a marked loss of dopaminergic neurons in the substantia nigra pars compacta, which causes dopamine deficiency in the striatum, and the presence of intracytoplasmic eosinophilic inclusions known as Lewy bodies (LBs), with α-synuclein-immunoreactive neuronal pathology in the remaining neurons.

Creutzfeldt–Jakob disease (CJD) is a fatal neurodegenerative condition that is clinically characterized by rapidly progressive dementia, myoclonus, and ataxia. In terms of its pathology, the brain takes on a spongy appearance with abnormal prion protein (PrP) deposition. CJD occurs across all human populations, with an incidence of about 1.5 cases per million individuals per year [2]. CJD principally occurs in the age range of 50–80 years, although it is likely that the disease is clinically overlooked in the population older than 80 years [2].

PD and CJD are clinically and neuropathologically distinct. Survival after a diagnosis of CJD is typically less than 1 year [2, 3], whereas that for PD may be 10–20 years [4, 5]. Here, we report a Japanese autopsy case of PD with a clinical course of 17 years who later developed rapidly progressive CJD. Recent advances in immunohistochemical methods have revealed a high frequency of multiple pathologies in single cases [6, 7], and our case adds to the literature on the co-occurrence of PD and CJD as well as that on multiple pathologies in neurodegenerative disorders.

## Case presentation

**Fig. 1:**
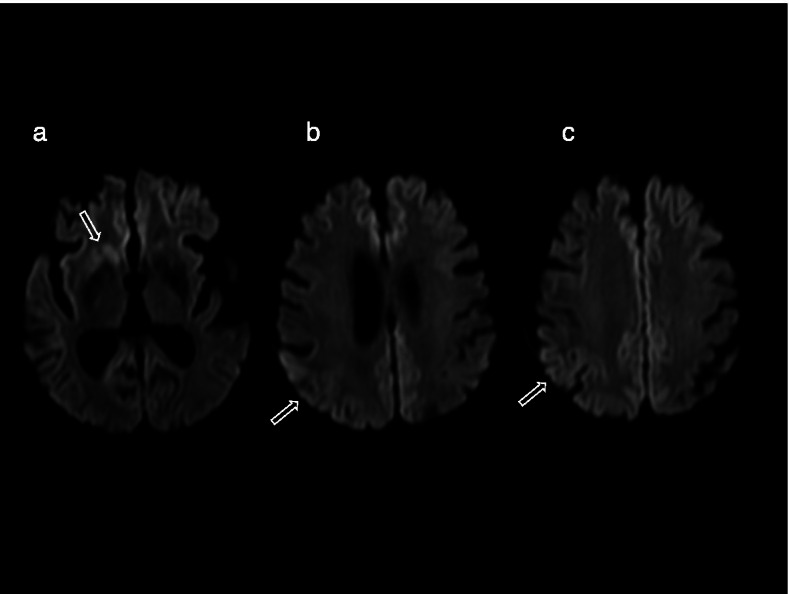
Diffusion-weighted magnetic resonance imaging from 4 months after the onset of memory impairment. The magnetic resonance images show hyperintense signals in the right lenticular and caudate nuclei (a) and right parietal cortex (b, c)

The patient was a Japanese woman who was 85 years old at the time of death. She had no family history of neurological diseases, including PD and CJD. She developed PD at the age of 68 years, with gradual left-sided upper extremity resting tremor, rigidity, bradykinesia, and difficulty with ambulation. Initial treatment with oral levodopa at 200 mg/day yielded an excellent response. Over a period of 8 years, because of progressive worsening of parkinsonism along with postural instability and freezing of gait, levodopa was gradually increased and associated treatment with ropinirole and droxidopa was added. The patient’s Hoehn and Yahr stage was III–IV until the age of 84 years, when she started developing memory impairment (in April 2019). The following month, she presented with visual hallucinations and delusions of theft despite the withdrawal of ropinirole and droxidopa and treatment with an acetylcholinesterase inhibitor. Her revised Hasegawa Dementia Scale [8] score was 12/30 in May 2019. In June 2019, myoclonus in the face and extremities were noted in addition to the exacerbation of generalized rigidity and bradykinesia; this led to nasogastric tube feeding. She had severe dementia and her Hoehn and Yahr stage was V, with no response to levodopa at 900 mg/day for 3 weeks. After a few months of unfavorable clinical evolution, the patient developed akinetic mutism in August 2019. Deep tendon reflexes were normal and no Babinski sign was elicited. Magnetic resonance imaging of the brain using diffusion-weighted imaging (MR-DWI) revealed extensive cortical ribbon-like and basal ganglia hypersignals, which were strongly consistent with CJD (Fig. 1). An electroencephalogram (EEG) was abnormal and showed diffuse slow waves with periodic synchronous discharges (PSD). Cerebrospinal fluid (CSF) examination [9] revealed elevation of 14-3-3 protein (>500 μg/mL) and total tau (>2,200 pg/mL). Real-time quaking-induced conversion (RT-QuIC) for PrP assays were positive using CSF [10]. The patient died of pneumonia in April 2020. The autopsy was limited to the brain.

### Neuropathological findings

**Fig. 2:**
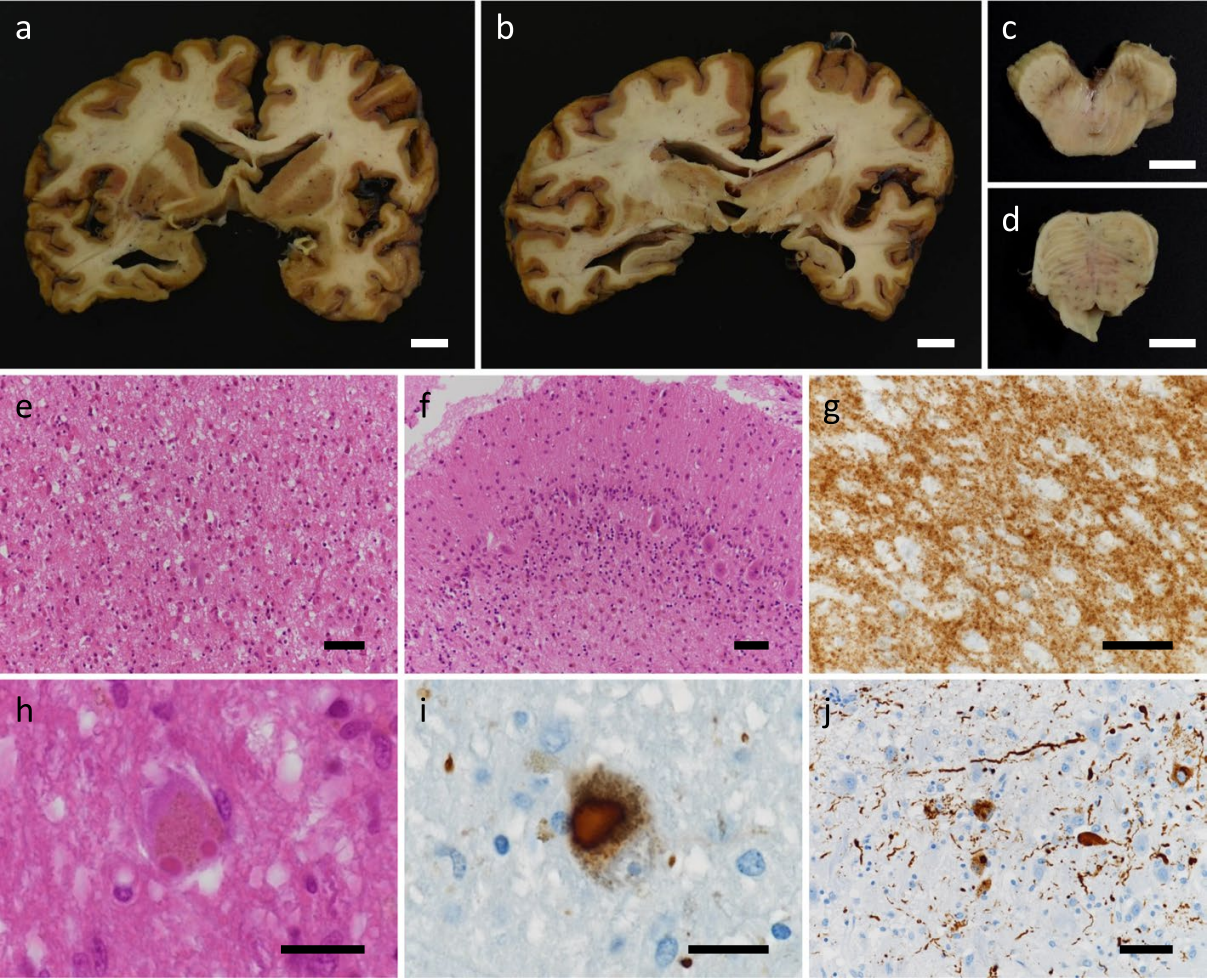
Histopathology. Diffuse marked cerebral atrophy (a, b) and depigmentation of the substantia nigra (c) and locus ceruleus (d) are shown. Post-mortem tissue staining of the frontal cortex (e, g), cerebellar cortex (f), Edinger–Westphal nucleus (h), substantia nigra (i), and cornu ammonis 2 (j) is also shown. (e, f, h) Hematoxylin and eosin staining; (g) prion immunostaining; (i, j) phosphorylated α-synuclein immunostaining. Scale bars: a–d, 1 cm; e–g, j, 50 μm; h, i, 20 μm)

An autopsy was performed 15 hours after death. The brain weighed 1,000 g before fixation. A detailed description of neuropathological examination methods is provided in Supplementary Information (Additional file 1). Macroscopic examination revealed severe diffuse atrophy of both the cerebrum and cerebellum (Fig. 2a, b). The hippocampus was relatively spared but mildly atrophic. The olfactory bulb showed thinning. Depigmentation was evident in the substantia nigra and locus ceruleus (Fig. 2c, d). Microscopic examination revealed widespread, severe neuronal loss and gliosis, with ballooned neurons in the cerebral cortex. Fine vacuole-type spongiform changes were detected in all layers of the cerebral cortex that were examined (Fig. 2e). The entorhinal and transentorhinal cortices showed severe spongiform changes and neuronal loss, whereas the hippocampus and subiculum showed minimal changes. The striatum and thalamus, particularly in the medial part, showed apparent spongiform changes. The globus pallidus and subthalamic nucleus were generally preserved. In the cerebellum, the molecular layer showed mild spongiform changes; neuronal loss was severe in the granule cell layer and mild to moderate in the Purkinje cell layer (Fig. 2f). In the brainstem, the locus ceruleus and substantia nigra showed severe neuronal loss and gliosis, while such changes were not apparent in the inferior olivary nucleus. Immunostaining for PrP revealed synaptic-type PrP deposition in all layers of the cerebral cortex (Fig. 2g) as well as in the striatum, thalamus, and cerebellar molecular and granular cell layers. The hippocampus, subiculum, globus pallidus, dentate nucleus, substantia nigra, and inferior olivary nucleus also showed very mild to mild synaptic-type PrP deposition. No plaque-type PrP deposition was observed in the regions mentioned above. PrP deposition was not observed in the cerebral or cerebellar white matter.

LB-related α-synucleinopathy (i.e., LBs, Lewy neurites, and neuronal intracytoplasmic inclusions that are immunoreactive for phosphorylated α-synuclein) was also distributed throughout the brainstem, including in the dorsal motor nucleus of the vagus nerve, locus ceruleus, substantia nigra, and Edinger–Westphal nucleus. It was also present in the nucleus basalis of Meynert and the limbic systems, including in the olfactory bulb, amygdala, hippocampus, and transentorhinal and cingulate cortices (Fig. 2h–j). Only a few phosphorylated α-synuclein-immunoreactive neuronal intracytoplasmic inclusions were observed in the neocortex.

Furthermore, scattered tufted astrocytes, globose-type neurofibrillary tangles, and coiled bodies that were immunoreactive for 4-repeat tau but not 3-repeat tau were observed in the inferior olivary nucleus, substantia nigra, red nucleus, superior colliculus, and periaqueductal gray matter of the midbrain, as well as in the subthalamic nucleus, putamen, globus pallidus, and precentral gyrus.

The level of Alzheimer’s disease-related neuropathological changes corresponded to “low” according to the National Institute on Aging-Alzheimer’s Association classification [11] (Thal phase for amyloid β plaques: 3 [12], Consortium to Establish a Registry for Alzheimer's Disease score: B [13], Braak NFT stage: II [14]). Very few phosphorylated TAR DNA-binding protein 43 (TDP-43)-immunoreactive neurites were observed in the uncus of the anterior hippocampus. No argyrophilic grains were identified.

### PrP gene polymorphism and western blot studies

**Fig. 3:**
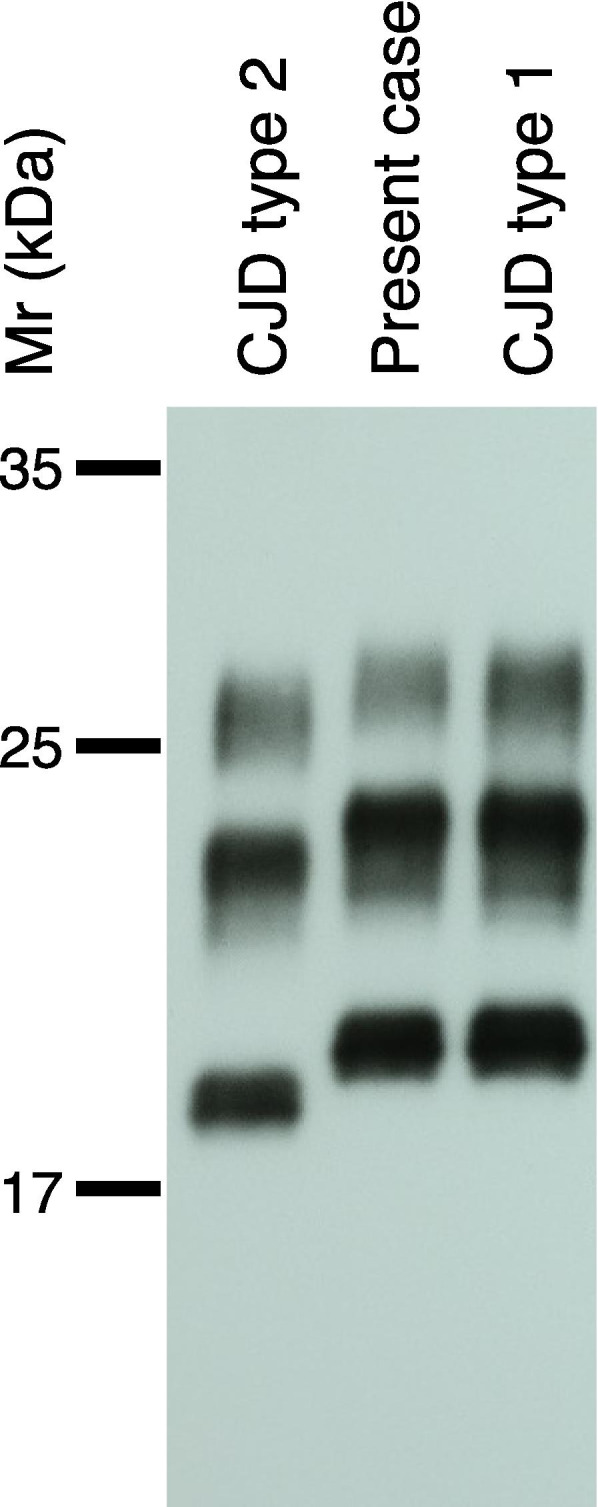
Western blot analysis of prion protein. The gel mobility of prion protein from the present case is compared with those from controls with Creutzfeldt–Jakob disease types 1 and 2

Gene analysis was done using genomic DNA extracted from the autopsied brain. PCR direct sequencing for the *PRNP* gene that encodes PrP revealed no mutations. The reference sequence used for the *PRNP* was NM_000311.5. Polymorphic codons showed methionine homozygosity at codon 129 and glutamate homozygosity at codon 219. Immunoblot analysis using anti-PrP antibody (3F4, Signet, Dedham, MA, USA) on a homogenized brain sample disclosed a type 1 pattern (Fig. 3; Supplementary Information for the original, unprocessed full-length version in Additional file 2).

## Discussion and conclusions

In the literature, the clinical and pathological features of six CJD patients with LBs have been described previously (Table 1) [15–20]. Although the clinical sequence of illness is variable in CJD, a change in mental status usually occurs before motor symptoms become apparent. Only one case has previously been reported in which PD preceded CJD [15]; the initial clinical diagnoses of the other five cases were Alzheimer’s disease [16], mood disorder [17], CJD [18], mild cognitive impairment [19], and visual hallucination/delusion [20]. Because the interval between PD onset and CJD onset in the previously reported case was 2.5 years, the present case is—to the best of our knowledge—the first report of a case of PD with a typical long clinical course who later developed neuropathologically confirmed CJD. Given that neuropsychiatric symptoms represent milestones of late stages of PD, usually occurring 10-15 years after onset of parkinsonism [21], the short interval of 2.5 years in the previously reported case would suggest a high possibility of coexisting neuropsychiatric disorders such as CJD. However, the long course of PD in our patient meant that the diagnosis of CJD was relatively difficult because her neuropsychiatric symptoms could also be regarded as non-motor symptoms of the later stages of PD [22]. Because changes in mental status are common to both of these conditions, identifying CJD in patients with late-stage PD can prove challenging. There have been no systematic attempts to estimate the incidence of their co-occurrence in single patients; however, assuming a prevalence rate of 130 per 100,000 for PD [1] and an incidence rate of 0.15 per 100,000 per year for CJD [2], their co-occurrence would be expected in two people per billion per year. Nevertheless, only two cases (including our patient; Table 1) have been reported to have developed CJD after an established clinical diagnosis of PD, suggesting that a number of such cases may simply go unreported or be overlooked. In contrast to the time interval between onset of neuropsychiatric symptoms and death in PD (3-5 years [5]), the interval is so short in CJD (< 1 year [2, 3]) that usually neither CJD patients nor their families have time to reflect, arrange affairs, or fully anticipate the disease progress and mortal outcome. Therefore, arriving at the correct diagnosis as early as possible would be helpful to patients and families. We hope that the present case report will encourage physicians to add CJD to their list of differential diagnoses for neuropsychiatric symptoms during the disease course of PD, particularly in its later stages. It is noteworthy that in a recent post-mortem series of sixteen cases with dementia with Lewy bodies (DLB) clinically suspected of CJD, the presence of MR-DWI hyperintensities and/or PSD on an EEG was more likely to distinguish CJD from DLB compared to that of clinical signs such as myoclonus or ataxia [23]. In this context, MR-DWI and EEG would be tests of choice for PD patients presenting with neuropsychiatric symptoms as well as myoclonus or ataxia. In fact, ataxia, one of the characteristic features of CJD [2], was clinically undetectable in our patient due to the severe dementia and akinesia in spite of the presence of pathologically extensive cerebellar lesions, while testing MR-DWI and EEG lead to the clinical diagnosis of CJD.

**Table 1:** Clinicopathological features of CJD cases with Lewy body disease in the literature

Author (published year)	Ezrin-Waters et al. (1985) [15]	Iida et al. (2001) [16]	Vital et al. (2007) [17]	Haraguchi et al. (2009) [18]	Fernández-Vega et al. (2015) [19]	Vita et al. (2017) [20]	Present case
Clinical diagnosis	PD + CJD	AD (or LBD) + CJD	CJD	CJD	CJD	CJD	PD + CJD
Pathological diagnosis	PD + CJD	DLB + CJD	CJD + AD + LBD	CJD + LBD + AD	CJD + LBD + AGD	CJD + LBD	PD + CJD
Sex	Female	Female	Male	Male	Male	Female	Female
Age at onset, years	68	64	76	77	77	77	68
Age at the diagnosis of CJD	71	67	78	77	78	78	84
Age at death, years	71	69	79	77	80	78	85
Total disease duration, years (duration from onset to mutism)	3 (3)	4 to 5 (2 to 3)	3 (ND)	0.4 (0.25)	2.3 (ND)	> 1 (> 1)	17 (17)
Initial symptoms	Akinesia, Shuffling gait, Resting tremor	Insomnia, Restlessness	Fluctuations in mood	Memory disturbance, Visual disturbance	Memory disturbance	Visual hallucination/delusion	Resting tremor, Rigidity, Bradykinesia, Difficulty with ambulation
Parkinsonism prior to diagnosis of CJD (age at onset of symptoms)	+ (68)	+ (65)	-	-	-	-	+ (68)
Neuropsychiatric symptoms prior to diagnosis of CJD (age at onset of symptoms)	-	+ (64)	± (76)	-	+ (77)	± (77)	-
Myoclonus	+	+	+	+	+	ND	+
Cerebellar signs	ND	ND	+	ND	ND	ND	-
Visual symptoms	+	ND	ND	+	ND	+	+
Pyramidal sign	+ (hyperreflexia/pathological reflex)	ND	ND	ND	+ (pathological reflex*)	-	-
EEG periodic synchronous discharges	+	+	+	+	+	+	+
CSF 14-3-3 protein	ND	+	+	+	-	+	+
MR-DWI hyperintensity of cerebral cortices and/or basal ganglia	ND	ND	ND	+	+	+	+
*PRNP* gene	ND	129MV; 219GG	129MV	129MM; 219GG	129MM	129MM	129MM; 219GG
PrP Western blotting	ND	ND	Type 1	Type 1	Type 2	Type 1+2	Type 1

AD: Alzheimer's disease; AGD: argyrophilic grain disease; CJD: Creutzfeldt-Jakob disease; CSF: cerebrospinal fluid; DLB: dementia with Lewy bodies; EEG: electroencephalogram; LBD: Lewy body disease; MR-DWI: magnetic resonance imaging-diffusion weighted image; ND: not described; PD: Parkinson's disease; PrP: prion protein

*Information from Iván Fernández-Vega. (personal communication, October 23, 2021)

Despite the usual limitations of cross-sectional assessments of end-stage neuropathology, we also found focal scattered tufted astrocytes, globose-type neurofibrillary tangles, and coiled bodies that were immunoreactive for 4-repeat tau in our patient; this corresponds to the early stages of progressive supranuclear palsy [24]. Notably, the coexistence of the deposition of proteins such as amyloid-β, tau, and/or TDP-43 in brains with CJD with LBs has been reported in five out of the seven published autopsy cases (including our patient; Table 1), thus emphasizing the importance of mixed pathologies in clinico-neuropathological studies of CJD as well as in older individuals. Indeed, it has been noted that the comorbidity of neurodegenerative diseases occurs more frequently than would be expected from the epidemiological data for each disease [25–27]. Molecular cross-talk among misfolded proteins through cross-seeding might explain the frequent finding of mixed pathologies [28]. Although a causative link between PD and CJD remains poorly understood, the possibility of cross-seeding cannot be discounted, warranting biochemical diagnoses such as quantification and RT-QuIC for α-synuclein and PrP [28]. In contrast, double immunohistochemistry for phosphorylated α-synuclein and PrP exhibited no explicit colocalization in the substantia nigra in our case (data not shown). Moreover, PrP was stained as the synaptic pattern in the brain regions examined as described above, whereas phosphorylated α-synuclein in intracellular structures including the cytoplasm and neurites. Elucidating whether the coexistence of proteinopathies in single patients is coincidental or not could aid to understand the etiology of the comorbidity of neurodegenerative diseases.

In conclusion, the present study demonstrated the clinicopathological co-occurrence of PD and CJD in a sporadic patient. The possibility of mixed pathology should be taken into account when neuropsychiatric symptoms develop, even in late-stage PD.

## Supplementary Information

**Additional file 1.** Procedures for neuropathological examination.

**Additional file 2.** Original, unprocessed version of immunoblot for PrP.

## Data Availability

The data that support the findings presented in this study are available from the corresponding author upon reasonable request.

## List of Abbreviations

CJD: Creutzfeldt–Jakob disease
LB: Lewy body
PD: Parkinson’s disease
PrP: prion protein
TDP-43: TAR DNA-binding protein 43

## References

[1] Pringsheim T, Jette N, Frolkis A, Steeves TD (2014). The prevalence of Parkinson's disease: a systematic review and meta-analysis. Mov Disord..

[2] LS. H. Prion diseases. In: Lewis ED MS, Rowland LP, eds, editor. Merritt’s Neurology, thirteenth ed. Pennsylvania: Lippincott Williams & Wilkins; 2015. p. 584–92.

[3] Parchi P, Giese A, Capellari S, Brown P, Schulz-Schaeffer W, Windl O, et al. Classification of sporadic Creutzfeldt-Jakob disease based on molecular and phenotypic analysis of 300 subjects. Ann Neurol. 1999;46(2):224–33. 10.1002/1531-8249(199908)46:2<224::AID-ANA12>3.0.CO;2-W. 10443888

[4] Nakashima K, Maeda M, Tabata M, Adachi Y, Kusumi M, Ohshiro H. Prognosis of Parkinson's disease in Japan. Tottori University Parkinson's Disease Epidemiology (TUPDE) Study Group. Eur Neurol. 1997;38(Suppl 2):60–3. 10.1159/000113485. 10.1159/000113485 9387805

[5] Kempster PA, O'Sullivan SS, Holton JL, Revesz T, Lees AJ (2010). Relationships between age and late progression of Parkinson's disease: a clinico-pathological study. Brain..

[6] Kapasi A, DeCarli C, Schneider JA (2017). Impact of multiple pathologies on the threshold for clinically overt dementia. Acta Neuropathol..

[7] Robinson JL, Lee EB, Xie SX, Rennert L, Suh E, Bredenberg C (2018). Neurodegenerative disease concomitant proteinopathies are prevalent, age-related and APOE4-associated. Brain..

[8] Hasegawa K (1974). An investigation of dementia rating scale for the elderly. Seishinigaku..

[9] Satoh K, Shirabe S, Eguchi H, Tsujino A, Eguchi K, Satoh A (2006). 14-3-3 protein, total tau and phosphorylated tau in cerebrospinal fluid of patients with Creutzfeldt-Jakob disease and neurodegenerative disease in Japan. Cell Mol Neurobiol..

[10] Atarashi R, Satoh K, Sano K, Fuse T, Yamaguchi N, Ishibashi D (2011). Ultrasensitive human prion detection in cerebrospinal fluid by real-time quaking-induced conversion. Nat Med..

[11] Montine TJ, Phelps CH, Beach TG, Bigio EH, Cairns NJ, Dickson DW (2012). National Institute on Aging-Alzheimer's Association guidelines for the neuropathologic assessment of Alzheimer's disease: a practical approach. Acta Neuropathol..

[12] Thal DR, Rub U, Orantes M, Braak H (2002). Phases of A beta-deposition in the human brain and its relevance for the development of AD. Neurology..

[13] Mirra SS, Heyman A, McKeel D, Sumi SM, Crain BJ, Brownlee LM, et al. The Consortium to Establish a Registry for Alzheimer's Disease (CERAD). Part II. Standardization of the neuropathologic assessment of Alzheimer's disease. Neurology. 1991;41(4):479-86. 10.1212/WNL.41.4.479. 10.1212/wnl.41.4.479 2011243

[14] Braak H, Braak E (1991). Neuropathological stageing of Alzheimer-related changes. Acta Neuropathol..

[15] Ezrin-Waters C, Resch L, Lang AE (1985). Coexistence of idiopathic Parkinson's disease and Creutzfeldt-Jakob disease. Can J Neurol Sci..

[16] Iida T, Doh-ura K, Kawashima T, Abe H, Iwaki T (2001). An atypical case of sporadic Creutzfeldt-Jakob disease with Parkinson's disease. Neuropathology..

[17] Vital A, Canron MH, Gil R, Hauw JJ, Vital C (2007). A sporadic case of Creutzfeldt-Jakob disease with beta-amyloid deposits and alpha-synuclein inclusions. Neuropathology..

[18] Haraguchi T, Terada S, Ishizu H, Sakai K, Tanabe Y, Nagai T (2009). Coexistence of Creutzfeldt-Jakob disease, Lewy body disease, and Alzheimer's disease pathology: an autopsy case showing typical clinical features of Creutzfeldt-Jakob disease. Neuropathology..

[19] Fernandez-Vega I, Ruiz-Ojeda J, Juste RA, Geijo M, Zarranz JJ, Sanchez Menoyo JL (2015). Coexistence of mixed phenotype Creutzfeldt-Jakob disease, Lewy body disease and argyrophilic grain disease plus histological features of possible Alzheimer's disease: a multi-protein disorder in an autopsy case. Neuropathology..

[20] Vita MG, Tiple D, Bizzarro A, Ladogana A, Colaizzo E, Capellari S (2017). Patient with rapidly evolving neurological disease with neuropathological lesions of Creutzfeldt-Jakob disease, Lewy body dementia, chronic subcortical vascular encephalopathy and meningothelial meningioma. Neuropathology..

[21] Hely MA, Reid WG, Adena MA, Halliday GM, Morris JG (2008). The Sydney multicenter study of Parkinson's disease: the inevitability of dementia at 20 years. Mov Disord..

[22] Coelho M, Ferreira JJ (2012). Late-stage Parkinson disease. Nat Rev Neurol..

[23] Geut H, Vergouw LJM, Galis Y, Ingrassia A, de Jong FJ, Quadri M (2019). Neuropathological and genetic characteristics of a post-mortem series of cases with dementia with Lewy bodies clinically suspected of Creutzfeldt-Jakob's disease. Parkinsonism Relat Disord..

[24] Nogami A, Yamazaki M, Saito Y, Hatsuta H, Sakiyama Y, Takao M (2015). Early Stage of Progressive Supranuclear Palsy: A Neuropathological Study of 324 Consecutive Autopsy Cases. J Nippon Med Sch..

[25] Forrest SL, Kim JH, De Sousa C, Cheong R, Crockford DR, Sheedy D (2021). Coexisting Lewy body disease and clinical parkinsonism in amyotrophic lateral sclerosis. Eur J Neurol..

[26] Araki K, Sumikura H, Matsudaira T, Sugiura A, Takao M, Murayama S (2016). Progressive supranuclear palsy and Parkinson's disease overlap: A clinicopathological case report. Neuropathology..

[27] Fujita K, Matsubara T, Miyamoto R, Sumikura H, Takeuchi T, Maruyama Saladini K, et al. Co-morbidity of progressive supranuclear palsy and amyotrophic lateral sclerosis: a clinical-pathological case report. BMC Neurol. 2019;19(1):168. 10.1186/s12883-019-1402-7. 10.1186/s12883-019-1402-7 PMC6637486 31319800

[28] Soto C, Pritzkow S (2018). Protein misfolding, aggregation, and conformational strains in neurodegenerative diseases. Nat Neurosci..

