# Supplementary material for: Parkinson’s disease with a typical clinical course of 17 years overlapped by Creutzfeldt–Jakob disease: an autopsy case report
Source: BMC Neurol. 2021 Dec 10;21:480. doi: 10.1186/s12883-021-02504-1 (PMC8662831; doi:10.1186/s12883-021-02504-1)
Supplement: Supplementary file 1 — Additional file 1. Procedures for neuropathological examination. [file 12883_2021_2504_MOESM1_ESM.docx]

**Additional file 1**

**Neuropathological Examination**

The brain was ﬁxed in 20% buffered formalin, and partially collected and frozen for protein analysis. The cerebrum, brainstem, and cerebellum were dissected in the coronal, axial, and sagittal planes, respectively. Representative anatomical areas were sampled, immersed in the formic acid for 1 hour to inactivate prion infectivity, and then embedded in parafﬁn. Six-μm-thick sections were stained with hematoxylin and eosin and Klüver–Barrera, and by Gallyas–Braak silver impregnation. Subsequently, immunoreaction product deposits on immunohistochemically stained sections were visualized with a Ventana BenchMark GX autostainer (Ventana Medical Systems, Tucson, AZ, USA), an I-View Universal DAB Detection Kit (Roche, Basel, Switzerland), and primary antibodies against prion protein (3F4; Covance, Princeton, NJ, USA) phosphorylated α-synuclein (pSyn#64; a gift from T. Iwatsubo, Japan; now available for purchase from FUJIFILM Wako Pure Chemical Corporation, Osaka, Japan), phosphorylated tau (AT8; Innogenetics, Ghent, Belgium), tau 4-repeat isoform RD4 (1E1/A6; Upstate, Lake Placid, NY, USA), tau 3-repeat isoform RD3 (8E6/C11; Upstate, Lake Placid, NY, USA), amyloid β (12B2; IBL, Gunma, Japan), and phosphorylated TDP43 (pSer409/410; a gift from M. Hasegawa, Japan; now available for purchase from Cosmo Bio, Tokyo, Japan). The specimen was investigated with a light microscope (Eclipse Ni, Nikon, Tokyo, Japan) and photographed using a digital camera (DS-Ri, Nikon, Tokyo, Japan).
