# Supplementary material for: Parkinson’s disease with a typical clinical course of 17 years overlapped by Creutzfeldt–Jakob disease: an autopsy case report
Source: BMC Neurol. 2021 Dec 10;21:480. doi: 10.1186/s12883-021-02504-1 (PMC8662831; doi:10.1186/s12883-021-02504-1)
Supplement: Supplementary file 2 — Additional file 2. Original, unprocessed version of immunoblot for PrP. [file 12883_2021_2504_MOESM2_ESM.pptx]

## Slide 1
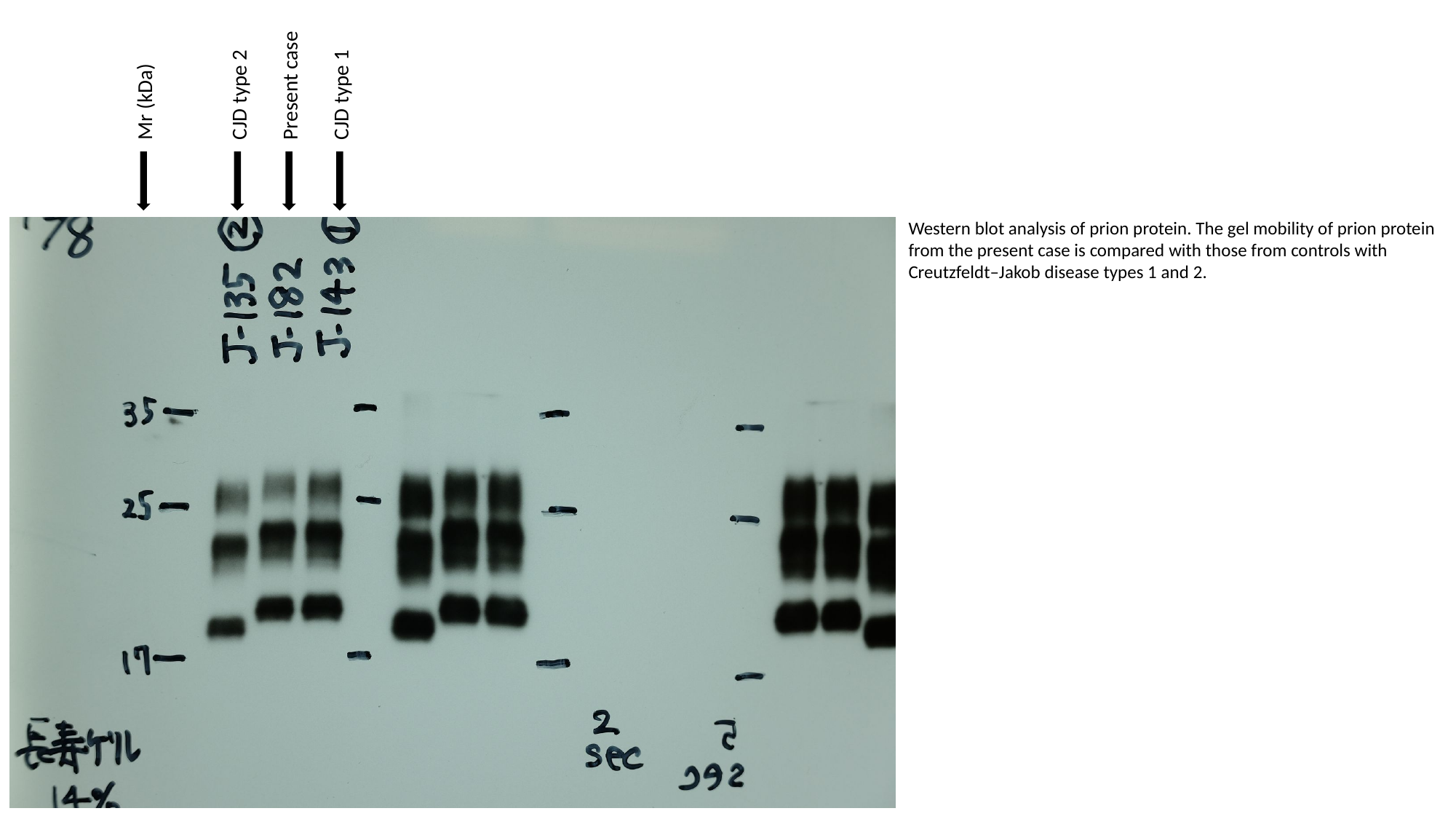

Mr (kDa)
CJD type 2
Present case
CJD type 1
Western blot analysis of prion protein. The gel mobility of prion protein from the present case is compared with those from controls with Creutzfeldt–Jakob disease types 1 and 2.
